# Non-haemorrhagic uterine rupture at 28 weeks of pregnancy following previous caesarean section: a case report

**DOI:** 10.1186/s12884-021-03990-4

**Published:** 2021-07-24

**Authors:** Alfred Lumala, Vicent Atwijukire

**Affiliations:** Kitovu Hospital, Masaka, Uganda

**Keywords:** Previous caesarean section, Severe oligohydramnios, Haemodynamically stable, Case report

## Abstract

**Background:**

There is need to put forward more symptoms and signs that could suggest a diagnosis of uterine rupture so that clinicians’ suspicion is increased; there is also need to put forward uncommon intraoperative findings in patients with uterine rupture to correlate with the signs and symptoms of patients.

**Case presentation:**

A 33 year old Gravida 5 Para 4 + 0 with 2 previous caesarean section scars at 28 weeks of amenorrhoea, presented to hospital complaining of lower abdominal pain for 11 h. She had no vaginal bleeding or vaginal discharge or pain on passing urine. On examination she had no pallor, pulse rate was 84 bpm, blood pressure was 110/80 mm of mercury (mmHg), fundal height was 27 cm (cm), fetal heart rate was regular at 150 beats per minute (bpm) and her cervix had a parous os. She was diagnosed with preterm labour and given dexamethasone intramuscularly, then an obstetric ultrasound scan was done and it revealed severe oligohydramnios. Decision do deliver her by emergency caesarean section was made and intraoperative findings were of a uterine rupture along the uterine scar with a fetal arm protruding through and vernix caseosa in the peritoneal cavity, without active uterine bleeding. The patient recovered well postoperatively.

**Conclusions:**

There is need to suspect uterine rupture in pregnant women with previous caesarean section scars if they present with abdominal pain and are found to have severe oligohydramnios despite having no history of any vaginal discharge, even when the fetal heart rate is normal and they are haemodynamically stable and without vaginal bleeding and remote from term.

## Background

Uterine rupture following previous caesarean section is a documented risk; its incidence among women with at least one prior caesarean section was found to be 0.5% [1]. This complication contributes to maternal and perinatal morbidity and mortality [2]. Perinatal outcomes after uterine rupture depend on the interval between diagnosis and delivery and hence early diagnosis and delivery decrease fetal morbidity and mortality [3]. However, the initial signs and symptoms of uterine rupture are not very specific and this makes early diagnosis difficult [3]. There is therefore need to put forward other symptoms and signs that could suggest this diagnosis so that clinicians’ suspicion is increased; there is also need to put forward uncommon intraoperative findings in patients with uterine rupture to correlate with the signs and symptoms of patients.

## Case presentation

This was a case of a 33 year old Gravida 5 Para 4 + 0 with 2 previous lower segment caesarean section scars, at 28 weeks of amenorrhoea using Naegele’s formula. She was a peasant farmer by occupation and her husband was a peasant farmer too. The indications for the previous caesarean sections were; cephalopelvic disproportion during the third pregnancy and then inadequate pelvis with one previous scar during the fourth pregnancy. She had two living children and these were the ones born by caesarean section after having lost the first two children during their perinatal stages. She had no family history of multiple pregnancy or hypertension or diabetes. She presented with lower abdominal pain for 11 h; the pain was vague in nature, non-radiating, relieved by lying down, exacerbated by walking, but not severe enough to affect daily activities. She had no vaginal bleeding or any vaginal discharge; she clarified that she had not had any flow of fluid from the vagina along her thighs. She had not any other gastrointestinal complaints or genitourinary complaints. We had no access to her antenatal care records because she had received antenatal care from another health unit, and she had not moved with her antenatal care documents; therefore we could not find out whether she had received medicines like NSAIDs that affect amniotic fluid volume during pregnancy. She had no pallor of mucous membranes, pulse rate was 72 bpm, blood pressure was 110/80 mmHg, had a subumbilical midline incision scar, fetal heart rate was regular at 150 bpm, cervix was thick and with a parous os. A decision to admit her due to possible preterm labour and concealed abruptio placenta was made, and she was given dexamethasone intramuscularly. Obstetric ultrasound scan was done and it revealed severe oligohydramnios with no measurable amniotic fluid pool; estimated gestation age was 29 weeks and 1 day and estimated fetal weight was 1300 g. There was not any placental abnormality. A diagnosis of severe oligohydramnios with 2 previous scars was made. A differential diagnosis of preterm premature rupture of membranes was also made as a possible explanation for the oligohydramnios. Her haemoglobin level was 11.1 g per decilitre (g/dl), white cell count was 11,010/ millilitre (ml), platelet count was 363,000/ml.

**Fig. 1:**
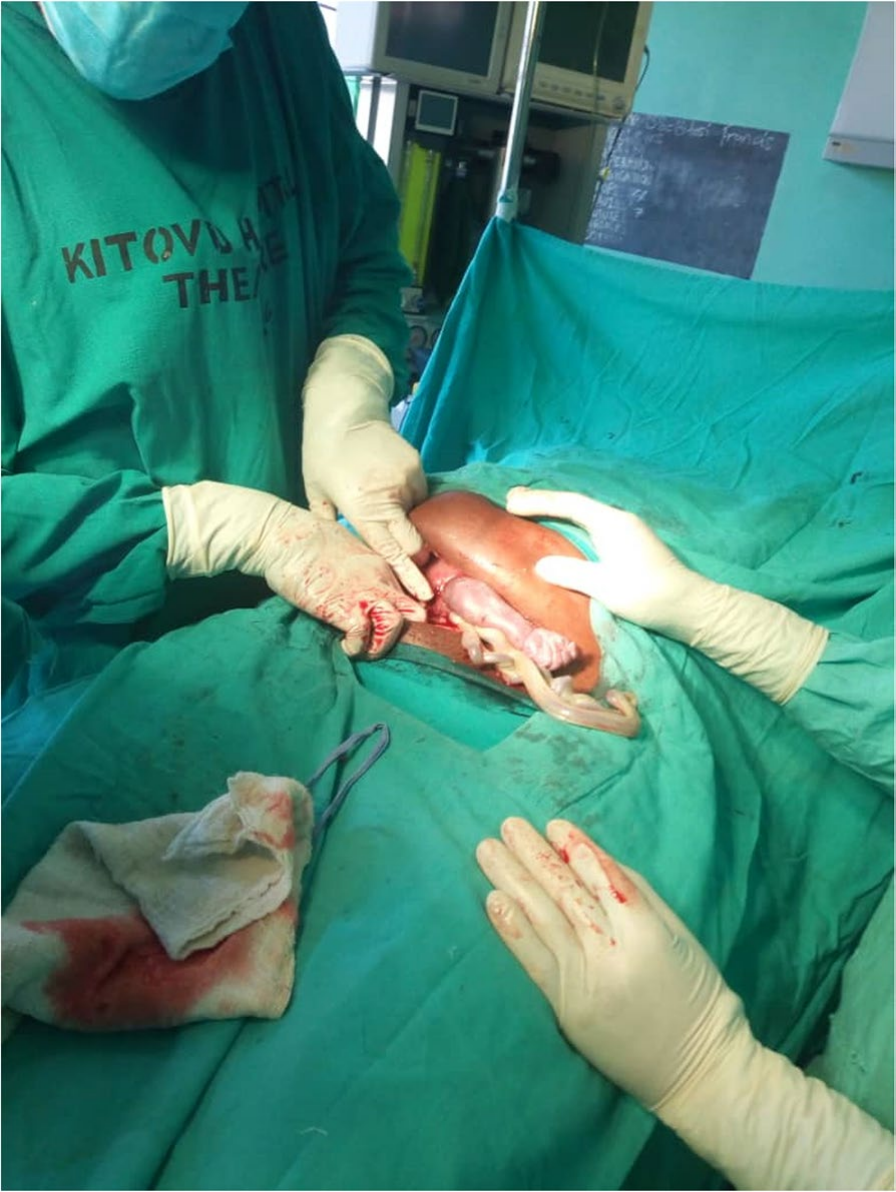
Intraoperative image

Decision was made to deliver her by emergency ceaesaren section; she received prophylactic antibiotics and intravenous fluids. Intraoperatively, we found a gravid uterus with a transverse rupture on the lower segment anteriorly and along the previous scar, about 6 cm long with a protruding fetal arm. There was no active bleeding from the uterus. The peritoneal fluid was observed to contain some vernix caseosa. A baby boy of apgar score 10 at 5 min and weight of 1200 g, was delivered and admitted to the neonatal intensive care unit, and there was no complication of the surgery with the patient recovering well after uterine repair. There were no postoperative investigations done because the patient recovered well. Bilateral tubal ligation was not done because she had not consented to it, but she got explanation about the risk of uterine rupture in a subsequent pregnancy and was given family planning counseling. Figure 1 showing the intraoperative findings and the timeline in Table 1 are attached to this case report article.

**Table 1:** Patient timeline

DAY OF CARE	TIME	ACTION ON PATIENT
DAY 1	5 AM	Admission of patient in hospital
	9 AM	Obstetric ultrasound scan done to reveal severe oligohydramnios
	12 Noon	Delivery by caesarean section and findings of uterine rupture noted
	1 PM	Routine postoperative care started and baby admitted to neonatal intensive care unit
DAY 4	9 AM	Patient discharged from hospital in good condition

## Discussion and conclusions

Uterine rupture during pregnancy is most common among women with previous caesarean section delivery, and while some cases are asymptomatic, the common symptoms are vaginal bleeding and abdominal pain and the commonest sign is fetal heart rate abnormalities [4]. However, it is possible that some cases of uterine are taken to be asymptomatic because the symptoms and signs they have, have not been put forward yet, and so this puts some cases at risk of poor outcomes through not being diagnosed in time. In this case, we had the advantage of an available team of clinical staff, but then found challenges in accessing obstetric ultrasound scan services soon enough; we also had not suspected uterine rupture at 28 weeks of amenorrhoea because this complication often happens at term. This case report shows that a patient did not have vaginal bleeding or fetal heart rate abnormalities, but had abdominal pain, and was later found to have severe oligohydramnios despite not having history of any vaginal discharge; caesarean section was done due to the severe oligohydramnios, and it was intraoperatively that it was discovered that the amniotic fluid had drained into the peritoneal cavity via a uterine rupture. The presence of amniotic fluid in the peritoneal cavity was evidenced by the presence vernix caseosa in the peritoneal fluid. At the time of making a diagnosis of severe oligohydramnios, there was not a clear cause of the lack of amniotic fluid, but only a suspicion that probably she had had preterm premature rupture of membranes with a trickle of amniotic fluid that she had not noticed. Uterine rupture had not been suspected because besides having a normal fetal heart rate, the patient was haemodynamically stable with a normal pulse rate and normal blood pressure. Therefore, a finding of severe oligohydramnios in a pregnant woman with previous caesarean section delivery should increase the suspicion of uterine rupture even when a pregnancy is remote from term.

## Data Availability

The datasets generated and/or analysed during the current study are not publicly available due to need to ensure privacy and confidentiality, but are available from the corresponding author on reasonable request.

## Abbreviations

mmHg: Millimetres of mercury
cm: Centimetres
bpm: Beats per minute
g/dl: Grams per decilitre
ml: Millilitre
